# Knee Extensor Muscle Strength to Measure the Ability of Five Times Sit to Stand Independently in Patients with Incomplete Spinal Cord Injury

**DOI:** 10.21315/mjms2022.29.5.8

**Published:** 2022-10-28

**Authors:** Lugkana Mato, Nanniphada Chankavee, Sugalya Amatachaya, Thiwabhorn Thaweewannakij

**Affiliations:** 1 School of Physical Therapy, Faculty of Associated Medical Sciences, Khon Kaen University, Khon Kaen, Thailand; 2 Improvement of Physical Performance and Quality of Life (IPQ) Research Group, Khon Kaen University, Thailand

**Keywords:** spinal cord injury, muscle strength dynamometer, falls, rehabilitation, reference value

## Abstract

**Background:**

Patients with incomplete spinal cord injury (iSCI) and lower extremity muscle weakness often fall while standing up from a chair. The sit-to-stand (STS) task primarily uses the strength of the knee extensor muscles. The five times sit-to-stand test (FTSST) is often applied to determine lower limb function and the results are related to lower extremity muscle strength. This study explored the cut-off point for knee extensor muscle strength in patients with iSCI to independently determine their FTSST results and the correlation between knee extensor muscle strength and FTSST results.

**Methods:**

Forty-four participants were assessed for knee extensor muscle strength using a hand-held dynamometer (HHD) and the FTSST.

**Results:**

The data indicated that knee extensor muscle strength ≥ 53.06 Newton was the best independent predictor of the FTSST results (sensitivity 72.7%, specificity 72.7%). Moreover, knee extensor muscle strength was significant and correlated with the FTSST results (*r* = −0.45, *P* = 0.035).

**Conclusion:**

The findings offer a cut-off point for the knee extensor muscle strength measured while standing up from a chair that may help medical professionals set rehabilitation goals for patients with iSCI.

## Introduction

Falls are a leading cause of injury and activity limitation among patients with incomplete spinal cord injury (iSCI). The adverse effects of falls result in significant individual, social and economic burdens. Approximately 34%–75% of ambulatory patients with iSCI have experienced at least one fall (1–4). According to previous studies, the factors associated with falls include environmental hazards (5, 6), the use of certain medicines (7), impaired balance and postural stability (8, 9) and poor lower extremity muscle strength (10, 11). As the cause of around 12%–33% of their falls, lower extremity muscle weakness (3, 12–14) is an important issue for patients with iSCI. One study found that patients with iSCI had a 32% incidence of falls when standing up from a chair, which is most likely due to the weakness of the lower extremity muscles and the knee extensor muscle in particular, as it is the primary muscle used to stand up (14).

The sit-to-stand (STS) movement is fundamental to normal activities. For instance, it is a pre-requisite for common daily movements, especially standing and walking (15, 16). The STS movement uses more force from the knee muscles relative to the hip, ankle and shoulder muscle groups (17). One previous study associated reduced force from the knee extensor muscles with difficulty standing up from a chair (18). Moreover, Wretenberg and Arborelius (19) observed that the knee extensor muscles contribute 72% of the force needed for the STS movement. Notably, the STS movement is commonly used to promote and assess lower limb function and mobility in individuals with impaired movement (20–23).

STS ability can be examined by recording either the number of stands during a given period or the timing of a given number of stands. However, the first method may cause muscle fatigue and soreness (24). The five times sit-to-stand test (FTSST) is often applied to assess functional lower extremity strength. This test is widely used and investigated due to its psychometric properties in many individuals, including the elderly (25, 26), children with cerebral palsy (27), stroke patients (28), patients with Parkinson’s disease (29) and patients with iSCI (30).

The most commonly used methods to assess muscle strength are the manual muscle test (MMT) and those involving a hand-held dynamometer (HHD). The MMT is the most commonly used method for measuring muscle strength in clinical assessments (31, 32). However, the MMT is a subjective measurement and ordinal scale, meaning that it depends on the experience and judgment of the examiner (31). In contrast, a HHD can measure the isometric contraction of a muscle (33). The reported value is given on a continuous scale. Crucially, a HHD is also portable and easy to use, with high to excellent levels of reliability (34, 35). Thus, the HHD method is superior to the MMT for detecting changes in muscle strength (36–38).

Evidently, knee extensor muscle strength is crucial in STS tasks. Therefore, a cut-off point for the knee extensor muscle strength used in STS tasks should help professionals set therapeutic goals for their patients, better preparing them to stand up and reducing the risk of falls. The current study investigated the cut-off point for the knee extensor muscle strength used during the STS movement to independently determine the FTSST results of patients with iSCI and explore the relationship between knee extensor muscle strength and FTSST results.

## Methods

### Participants

This assessor-blinded cross-sectional study was conducted in patients with non-traumatic and traumatic iSCIs (The American Spinal Cord Injury Association Impairment Scale [AIS Grade D]) (39). The participants were recruited from a tertiary rehabilitation centre and community hospitals in northeast Thailand using a simple random sampling method. The inclusion criteria were as follows: an iSCI at a chronic stage of injury (≥ 12 months after SCI) (14, 40), an age of at least 18 years old, a body mass index (BMI) from 18.5 kg/m^2^–29.9 kg/m^2^ and the ability to stand up from a chair five times independently with or without hand support (41). The exclusion criteria were spasticity greater than Grade 2 as measured by the Modified Ashworth Scale (MAS) (42), a leg-length discrepancy > 1.4 cm (43), deformity of the musculoskeletal system (41) and musculoskeletal pain with an intensity of pain > 5/10 on a numerical pain rating scale (44). The sample size was calculated using data from the pilot study (power level = 0.8 and significance level = 0.05). According to the results, the study required at least 44 subjects: 22 subjects who could stand up with hand support (the with hand support group) and 22 subjects who could stand up without hand support (the without hand support group). Prior to being enrolled in the study, all participants signed an informed consent form approved by the local ethics committee of Khon Kaen University (HE 591484).

### Protocol

The eligible patients participated in this study for two consecutive days. On the first day, the first examiner interviewed them to collect their demographic data and SCI characteristics—including cause, level of injury and post-injury time—and determined their FTSST results. On the second day, the second examiner measured their knee extensor muscle strength with a HHD.

### Five Times Sit-to-Stand Test

The participants in the without hand support group sat on a standard armless chair with their backs upright at a 90° angle against the backrest, their feet on the floor with their heels 10 cm behind their knees (41, 45) and their arms at their sides (46). The participants in the with hand support group sat in a similar manner but pressed their hands down against a standard walker. The first examiner manually began a digital stopwatch at the ‘Go’ instruction and stopped it when each participant’s back patted the backrest after their fifth stand (47–49). The test was conducted with three trials and the participants could rest for 5 min or longer between each trial depending on their fatigue (48). The mean value of the three trials was used for data analysis.

The criteria for classifying the FTSST results were based on the abilities of the participants. If the participants could not stand up five times without using the upper extremity, they were assigned to the with hand support group. If the participants could stand up five times continuously without using the upper extremity, they were assigned to the without hand support group. The present study reported excellent intra-(intraclass correlation coefficient [ICC] = 0.99) and inter-rater reliability for the FTSST (ICC = 0.98).

### Knee Extensor Muscle Strength Test

The participants used the passive movement of the lower extremity to normalise their muscle tone before the test. Each participant laid on their side with a knee flexion of approximately 90°, which exerts maximum force against the stationary HHD without any movement. The participants performed three trials on both sides. The rest period between trials was either 1 min to prevent fatigue or until each participant’s fatigue had disappeared (35). The HHD values were normalised according to the body weight of each participant. The peak forces were recorded in pounds and converted into Newtons. The average values of both sides were used for data analysis. The present study found excellent intra- (ICC = 0.98) and inter-rater reliability for the knee extensor muscle strength test (ICC = 0.97).

### Data Analysis

Descriptive statistics were used to explain the baseline demographics, iSCI characteristics and findings. The Shapiro-Wilk test was conducted to estimate the normality of the data. Receiver-operating characteristic (ROC) curves were applied to explore the optimal cut-off score, sensitivity, specificity and area under the receiver characteristic curve (AUC) of the knee extensor muscle strength values (50, 51). Independent *t*-tests and Mann-Whitney U tests were used to compare the data between groups with normal and non-normal distributions, respectively. Pearson’s correlation coefficient (*r*) was utilised to explain the correlation levels between the FTSST results and the knee extensor strength data. The strength of the correlation was defined as either mild (*r* = 0.30–0.50), moderate (*r* = 0.50–0.70) or strong (*r* = 0.70–0.90) (52). Statistical significance was set to *P* < 0.05.

## Results

A total of 44 participants with iSCI were included in this study: 22 participants in the hand support group and 22 participants in the without hand support group. Most participants were male (70%), had a non-traumatic injury (57%) and had a low level of injury (e.g. lumbar). Table 1 shows the demographics and SCI characteristics of the participants and Table 2 displays the knee extensor muscle strength data and FTSST results. Following the data, the without hand support group performed better than the with hand support group (*P* < 0.05).

**Figure 1:**
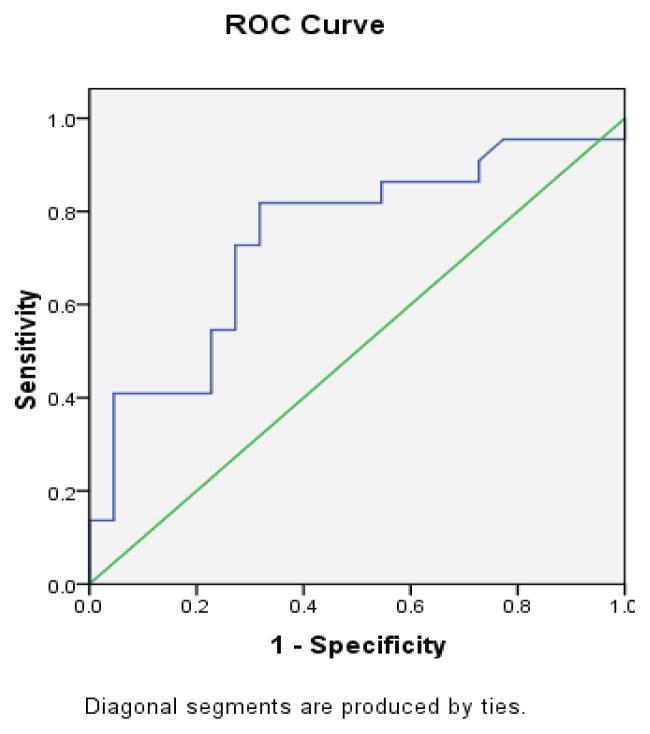
The receiver-operating characteristic curves of sensitivity and specificity of knee extensor muscle strength for determining the ability of FTSST independently without hands support

The data from the ROC curve indicated that the use of knee extensor muscle strength ≥ 53.06 Newtons could satisfactorily determine the FTSST results of the without hand support group (sensitivity = 72.7%, specificity = 72.2% and AUC = 0.74) (Figure 1). We found a significant negative correlation between the time that the without hand support group needed to complete the FTSST and the strength of the knee extensor muscle (*r* = −0.45, *P* = 0.0035) (Table 3).

## Discussion

This study investigated the cut-off point for knee extensor muscle strength to independently determine the ability of patients with iSCI to stand up five times from a chair without using their hands for support. This study found a correlation between knee extensor muscle strength and the FTSST without hand support group.

Multiple falls occur more frequently in patients with iSCI due to lower extremity muscle weakness. Approximately 32% of patients with iSCI fall while standing from a chair (14). This is explained by the STS movement entering the extension phase (phase III of STS), which is mechanically distinct from both the flexion-momentum and momentum transfer phases. In the extension phase, the body is transferred vertically while in an inherently stable position (53). Therefore, knee extensor muscle strength is essential for moving the body into an upright position (54).

Despite its importance, few studies have reported the cut-off point for knee extensor muscle strength relative to the STS movement. Using a HHD, Bohannon (55) reported a cut-off point of 330 Newton for knee extension force and a cut-off MMT score of 22 to independently determine if the elderly could stand up from a chair without hand support. Similarly, Eriksrud and Bohannon (56) obtained a cut-off point of 300 Newton for the total force using a HHD and a cut-off MMT score of 20.5 to independently predict whether acute rehabilitation patients could stand up from a chair. However, no previous study has focused on SCI patients. Furthermore, Eriksrud and Bohannon (56) did not normalise the data with the body weights of the participants, who also did not have muscle weakness problems. In our study, we found that a cut-off point of 53.06 Newton independently determined the ability of patients with iSCI to stand up from a chair without hand support.

When the participants supported themselves with their hands and performed the STS movement, we observed a 50% reduction in extension momentum at the knee joints. This also resulted in reduced muscle activation in the lower extremity muscles (57–59). Following the results, knee extensor muscle strength did not correlate with the FTSST results of the with hand support group. While standing up from a chair, the participants in this group used their arms to press their hands against an assistive device. Thus, they used this device more than their own legs while standing due to their knee extensor muscle weakness. The use of the upper limb while completing the STS movement affects the validity of FTSST results, which are supposed to represent lower extremity muscle function (30). Moreover, this study reported a correlation between knee extensor muscle strength and the FTSST results of the without hand support group. These results are like those of previous studies on elderly patients with cerebral palsy and acute rehabilitation patients (27, 54, 56).

This study had some limitations that must be considered when interpreting its findings. First, this study did not determine the strength cut-off points for other lower extremity muscles. Future studies should investigate these cut-off points to independently determine the FTSST results of patients with iSCI. Second, this study only included patients with AIS Grade D injuries. Consequently, the results cannot be generalised to injuries with different severities. Third, this study did not measure the force of the participants’ hands while they stood up from the chair. Hence, future studies should determine the force of the participants’ hands when they use their hands for support during the FTSST. Fourth, the sample size was small. Although the number of participants needed for the study was calculated before conducting the study, the effect size was still medium (60). The use of more participants in future studies will address this issue.

## Conclusion

This study identified the cut-off point for the knee extensor muscle strength needed to independently determine the ability of patients with iSCI to stand up from a chair five times without hand support and explored the correlation between knee extensor muscle strength and FTSST results. The findings suggest that when measured with a HHD, knee extensor muscle strength ≥ 53.06 Newton indicates that patients with iSCI may be able to stand up from a chair independently. These findings might help medical professionals set goals related to knee extensor muscle strength for patients with iSCI before they stand up.

## Figures and Tables

**Table 1:** Demographics and spinal cord injury characteristics of the participants

Variable	Total number of subjects (*n* = 44)	Groups	

		FTSST with hands (*n* = 22)	FTSST without hands (*n* = 22)
Age (years old) a	50.93 (15.88) (46.10, 55.76)	51.36 (18.12) (43.33, 59.40)	50.50 (13.70) (44.42, 56.58)
Body mass index (kg/m^2^) a	23.35 (4.20) (22.08, 24.63)	23.61 (4.01) (21.84, 25.39)	23.09 (4.46) (21.12, 25.07)
Post injury time (months) a	73.14 (64.72) (53.46, 92.81)	62.73 (68.66) (32.28, 93.17)	83.55 (60.29) (56.81, 110.28)
Genders: female/male b	13/31	6/16	7/15
Causes of SCI: traumatic/non-traumatic b	19/25	8/14	11/11
Levels of injury b: Cervical	9	3	6
Thoracic	12	9	3
Lumbar	18	8	10
Sacral	5	2	3
Spasticity by Modify Ashworth scale: b	1	0	1
Grade 0	17	8	9
Grade 1	26	14	12
Grade 2			

Notes:

a Data are presented using mean (SD) (95% confidence interval);

b Data are presented using number (*n*)

**Table 2:** Knee extensor muscle strength and FTSST of the participants

Variables	With hands support (*n* = 22)	Without hands support (*n* = 22)	*P*-value
Knee extensor muscle strength (N)	48.27 (13.22) (42.41, 54.13)	60.75 (16.47) (53.45, 68.06)	0.006¥
FTSST (s)	14.52 (2.82) (13.27, 15.77)	12.36 (3.24) (10.92, 13.80)	0.023€

Notes: Data are presented using mean (SD) (95% confidence interval);

€ *P*-value were analysed by using independent *t*-test;

¥ *P*-value were analysed by using Mann-Whitney U test;

N = Newton; s = seconds

**Table 3:** Correlations between FTSST with or without hands and knee extensor muscles strength

Variables	Knee extensor muscle strength	*P*-value
FTSST (with hands)	0.11	0.636
FTSST (without hands)	−0.45	0.035*

Notes:

* The data were analysed using the Pearson’s correlation coefficient with the *P*-value < 0.05;

FTSST = five times sit-to-stand test
